# Sustained and targeted episcleral delivery of celecoxib in a rabbit model of retinal and choroidal neovascularization

**DOI:** 10.1186/s40942-018-0131-1

**Published:** 2018-08-09

**Authors:** Luiz H. Lima, Michel E. Farah, Glenwood Gum, Pamela Ko, Ricardo A. de Carvalho

**Affiliations:** 1 Federal University of Sao Paulo, Rua Botucatu, 821, Vila Clementino, São Paulo, SP CEP: 04023-062 Brazil; 2 Biological Test Center, Irvine, CA USA; 3 3T Ophthalmics, Irvine, CA USA

**Keywords:** Celecoxib, Choroidal neovascularization, Episcleral delivery, Matrigel-induced model, Retinal neovascularization

## Abstract

**Background:**

To evaluate the efficacy of selective episcleral delivery of celecoxib formulated in a sustained-release episcleral exoplant on a model of retinal and choroidal neovascularization induced in rabbits by subretinal injection of matrigel combined with vascular endothelial growth factor (VEGF) and basic fibroblast growth factor (bFGF).

**Methods:**

Nine New Zealand white rabbits were randomly assigned to three groups (episcleral celecoxib exoplant, intravitreal bevacizumab injection and control group). The bFGF was mixed with matrigel at a concentration of 10 ug/0.1 mL, and VEGF was mixed with matrigel at a concentration of 2 ug/0.1 mL. Animals assigned to celecoxib or intravitreal bevacizumab groups were treated within 03 days from matrigel injection. Fluorescein angiography (FA) and electroretinography (ERG) were performed 5 days, 2, 4 and 8 weeks after matrigel injection. Persistence or regression of three clinical features (subretinal hyperfluorescence, retinal vascular tortuosity and retinal fibrotic spots) was independently evaluated in each study group at all follow-up periods. Statistical analysis using Fisher’s exact test was performed to compare the frequency of findings at each time point between treated groups and control.

**Results:**

In all study eyes, matrigel induced the appearance of subretinal blebs and the development of retinal and subretinal neovascularization characterized by progressive and late hyperfluorescence on FA. Persistence of subretinal hyperfluorescence was higher in non-treated (control) animals compared to celecoxib (*p* = 0.0238) treated animals. The mean b-wave amplitude ratios of ERG recordings did not reveal statistically significant differences between the study groups. Control animals retained in average 40% (± 7%) of the pre-treatment recorded b-wave amplitude, compared to 53% (± 29%) after bevacizumab and 53% (± 17%) after celecoxib treatment.

**Conclusion:**

In this rabbit model of retinal and subretinal neovascularization, episcleral celecoxib delivery was demonstrated to significantly inhibit neovascularization. It was also noticed, although not statistically significant, an apparent effect of episcleral celecoxib on preventing tractional retinal detachment secondary to epiretinal fibrovascular proliferation. The transscleral delivery of celecoxib combined with sustained-release strategy may have impact in the treatment of retinal and choroidal proliferative diseases.

## Background

Angiogenesis is an essential organic mechanism represented by the development of new vessels from preexisting blood vessels, and comprises the incitement of angiogenic growth factor receptors on vascular endothelial cells, migration and proliferation of endothelial cells and endothelial cell basal membrane breakdown [1, 2]. Vascular endothelial growth factor (VEGF) is an active cytokine that modulates the angiogenesis, stimulates retinal and choroidal new vessels growth and is considered crucial for the development of choroidal neovascularization (CNV) in neovascular age-related macular degeneration (AMD) patients [3, 4]. The establishment of VEGF as a critical regulator of AMD angiogenesis changed the field by stimulating the anti-VEGF drugs development and, therefore, inhibiting angiogenesis. Aflibercept, bevacizumab and ranibizumab are commercially usable antibodies that inhibit the VEGF isoforms activity, and its intravitreal use has been related to the improvement of visual acuity in eyes with neovascular AMD [5–7]. Recently, celecoxib, a selective cyclooxygenase-2 (COX-2) inhibitor, is reported to reduce diabetes-induced retinal VEGF expression, inhibition of proliferation of adult retinal pigment epithelial (ARPE-19) and choroidal endothelial cells [8, 9].

Although intravitreous injections permit very quick drug delivery to both retina and choroid, they lead to high peak of drug levels isolated by depressions that could be below the therapeutic threshold and also may increase the risk of retinal detachment and endophthalmitis [10]. Therefore, the current intravitreal injection strategy does not represent an ideal intraocular delivery profile for most therapeutic agents. The development of new intraocular routes of drug delivery that allows local delivery in ways that are less invasive and provide constant therapeutic drug levels in the retina and choroid may provide major benefits [10]. Transscleral drug delivery using an episcleral exoplant attached to the eye has been demonstrated to be safe and may increase the bioavailability of tracers in the posterior segment of the eye [10]. The purpose of this study was to evaluate the efficacy of celecoxib formulated in sustained-release poly-lactide-glycolide copolymers and delivered from silicone episcleral exoplants on a matrigel-based model of retinal and choroidal neovascularization in rabbits eyes.

## Methods

### Treatment groups and assessments

All the experiments were performed in accordance with the ARVO Statement for the Use of Animals in Ophthalmic and Vision Research. Nine female New Zealand rabbits with weight ranging from 2.0 to 4.0 kg were randomly assigned to three groups (celecoxib exoplant, intravitreal bevacizumab injection or control group).

In the present study, retinal and subretinal neovascularization was induced in all rabbits (*n* = 9) by subretinal injection of matrigel mixed with recombinant human basic fibroblast growth factor (bFGF) and vascular endothelial growth factor (VEGF). The bFGF was mixed with matrigel at a concentration of 10 ug/0.1 mL, and VEGF was mixed with matrigel at a concentration of 2 ug/0.1 mL. The mixture was made under a laminar flow hood and kept under sterile conditions. Matrigel was kept on ice throughout the procedure. The subretinal injection of matrigel was performed in the right eye of each animal. After peritomy and sclerotomy a small core vitrectomy was performed to facilitate the volume expansion of the bleb created by the subretinal injection. A cannula connected to a tuberculin syringe was inserted into the subretinal space and the volume of approximately 10–20 uL was delivered. The sclerotomy was closed with a 7-0 vicryl. Prior to surgical procedure, the animals were weighed and anesthetized with an intravenous injection of a ketamine/xylazine cocktail (87 mg/mL of ketamine and 13 mg/mL of xylazine) at a volume of 0.1–0.2 mL/kg.

**Fig. 1:**
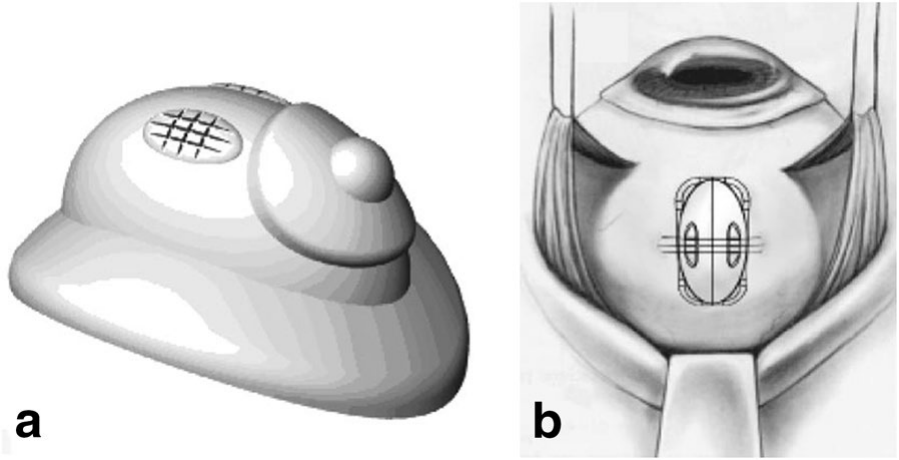
Schematic of episcleral device. **a** The appearance of poly (lactic-co-glycolic) acid (PLGA) episcleral exoplant. **b** The episcleral device attached to the globe

In addition to the matrigel-GF injection, in the celecoxib group (*n* = 3), rabbits had a poly (lactic-co-glycolic) acid (PLGA) device with a dose of 9.0 mg of celecoxib sutured to the sclera of the right eye to provide tight device apposition to the sclera (Fig. 1). In the bevacizumab group (*n* = 3), 0.05 mL (1.25 mg) of bevacizumab solution (Avastin^®^, Roche Genentech, South San Francisco, CA) was injected intravitreally in the right eye. In the control group (*n* = 3), the rabbits were submitted to 0.05 mL injection of phosphate buffered saline (PBS) in the right eye. The control group was represented by rabbits in which the matrigel-based model of retinal neovascularization was induced by not treated.

External ocular examinations, indirect ophthalmoscopy, color fundus photograph (Fundus Camera, TRC, Topcon, Japan), fluorescein angiography (FA) (Fundus Camera, TRC, Topcon, Japan) and electroretinography (ERG) were performed on both eyes of each animal at 5-day, 2-week, 4-week and 8-week follow-ups. Prior to FA, the animals were weighed and anesthetized following the same protocol described above. A rapid intravenous bolus injection of 0.3 mL of 10% fluorescein sodium solution was administered to the ear vein of each animal. Color fundus photographs were taken before the FA. Full-field scotopic and photopic ERG (Diagnosys LLC, Lowell, MA, USA) was performed according to the International Society for Clinical Electrophysiology of Vision (ISCEV) standards [11].

### Surgical procedures

For implantation of episcleral celecoxib exoplant, a conjunctival incision was made and the surface of the sclera was exposed and cleaned. Two episcleral polyester sutures (6-0 Ti-Cron, Syneture, Norwalk, CT, USA) were placed on each side of the device, and a drop of cyanoacrylate adhesive was applied to each corner of the device to hold it in place while the sutures were secured. Sutures were tied tightly to cause tight apposition of the exoplant to the sclera. The conjunctiva was closed over the exoplant with an absorbable suture (Polysorb, Syneture, Norwalk, CT, USA). NPB ophthalmic ointment was used immediately after exoplant implantation. For bevacizumab and placebo intravitreal injections, a 30-gauge needle connected to a tuberculin syringe was used. The needle was inserted into the vitreous cavity at approximately 2.0 mm from the limbus. NPB ophthalmic ointment was used immediately following injection.

### Data and statistical analyses

The persistence or regression of three clinical features related to the choroidal neovascularization developed after matrigel subretinal injection (subretinal hyperfluorescence, retinal vascular tortuosity and retinal fibrotic spots) were independently evaluated in each study group (transscleral celecoxib exoplant, intravitreal bevacizumab injection and control group) at all follow-up periods by two of the authors (LL and MF) who were masked to the other findings and data of the study rabbits. In the event of disagreement, a third investigator (RC) was consulted for the final determination. The development of tractional retinal detachment at 8-week follow-up was also evaluated in the study groups.

Statistical analysis was performed with commercial software (WinNonlin Pro 4.1; Pharsight Corp., Mountain View, CA, USA). Fisher’s exact test was used for multiple comparisons of clinical features between the three study groups (episcleral celecoxib exoplant, intravitreal bevacizumab injection and control group). A paired *t* test was used for two-sample comparisons of mean b-wave amplitude ratios of ERG recordings between the right and left eyes. Type I error was set below 0.05 for statistical significance.

## Results

**Fig. 2:**
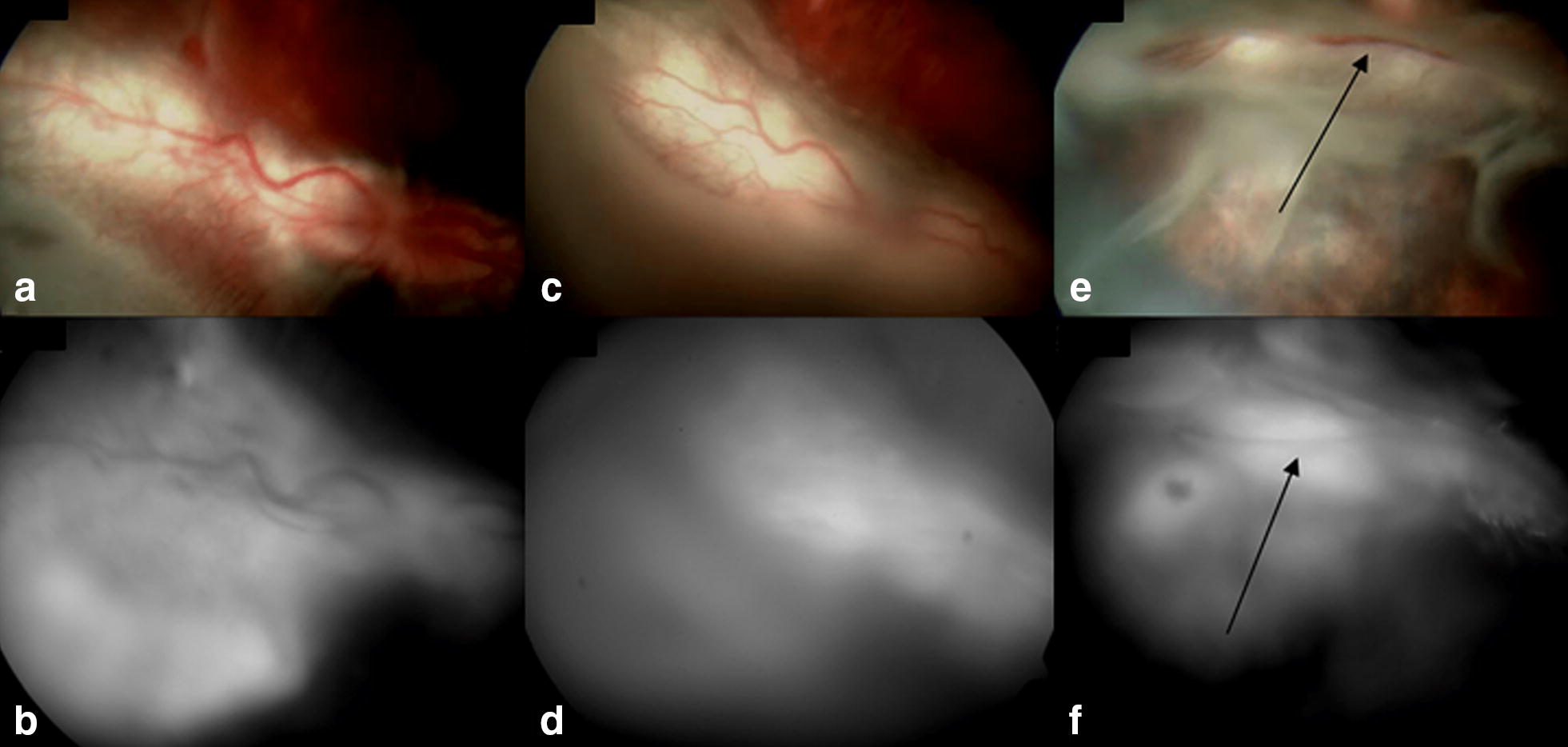
Study rabbit from control group. **a**, **b** Color photograph and fluorescein angiography (FA) show delineation of subretinal blebs immediately after subretinal injection of matrigel in the right eye. **c**, **d** At 2-week follow-up, retinal/choroidal neovascularization and focal retinal degeneration are observed in the fundus of right eye. **e**, **f** Persistence of subretinal hyperfluorescence, retinal vascular tortuosity and retinal fibrotic spots (arrows) are noticed in the right eye at 8-week follow-up examination

**Fig. 3:**
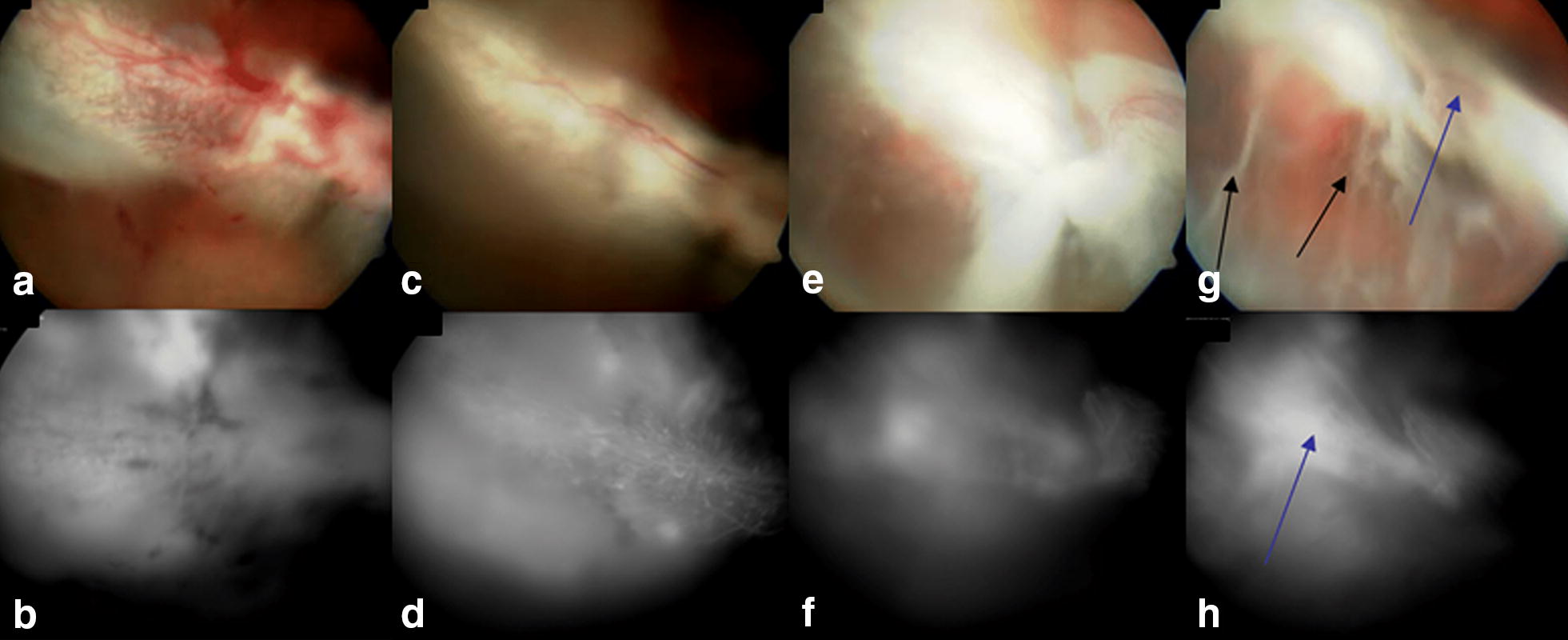
Study rabbit from intravitreal bevacizumab injection group. **a**, **b** Color photograph and FA show delineation of subretinal blebs immediately after subretinal injection of matrigel in the right eye. **c**, **d** At 2-week follow-up, retinal/choroidal neovascularization and focal retinal degeneration are observed in the fundus of right eye. **e**, **f** Persistence of subretinal hyperfluorescence is noticed in the right eye at 4-week follow-up examination. **g**, **h** At 8-week follow-up, an extensive tractional retinal detachment is detected in the right eye (arrows)

**Fig. 4:**
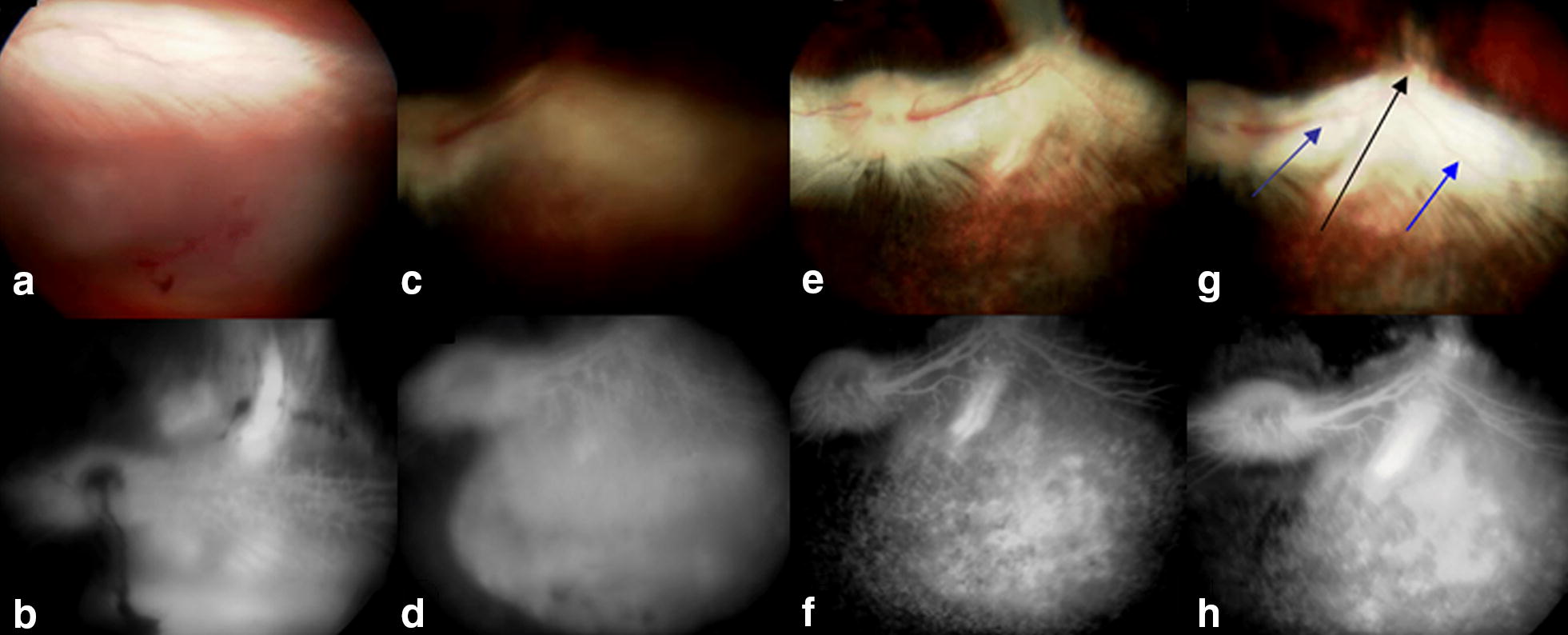
Study rabbit from transscleral celecoxib exoplant group. **a**, **b** Color photograph and FA show delineation of subretinal blebs immediately after subretinal injection of matrigel in the right eye. **c**, **d** At 2-week follow-up, retinal/choroidal neovascularization and focal retinal degeneration are noticed in the fundus of right eye. **e**, **f** Complete regression of subretinal hyperfluorescence, retinal vascular tortuosity and retinal fibrotic spots are observed in the right eye at 8-week examination. **g**, **h** Cicatricial retinal traction (arrows) is observed at 8-week follow-up examination

In all study eyes, the matrigel induced delineation of subretinal blebs immediately after its subretinal injection, and stimulated local angiogenesis resulting in localized retinal and subretinal neovascularization (Figs. 2, 3 and 4). The most common funduscopic and angiographic findings were increased tortuosity of the retinal vasculature, and subretinal and retinal hyperfluorescence that were seen in virtually all the animals within 1 week from matrigel injection. In the celecoxib exoplant group, there was progressive regression of these retinal features throughout the follow-up period with complete regression at 8-week examination. After intravitreal bevacizumab injection, persistence of subretinal hyperfluorescence was observed in two rabbits, and an extensive tractional retinal detachment occurred in one rabbit at 8-week follow-up. Cicatricial retinal detachment was observed in one rabbit from the episcleral exoplant group at 8-week follow-up examination (Figs. 2, 3 and 4).

Statistical analyses of study clinical features showed that persistence of subretinal hyperfluorescence, retinal vascular tortuosity and retinal fibrotic spots was higher in non-treated (control) animals compared to celecoxib (*p* = 0.0238) treated animals. Tractional retinal detachment development at 8-week follow-up was greater in the control and bevacizumab groups in comparison with celecoxib group, but without statistical significance (*p* = 0.1071) (Table 1). The mean b-wave amplitude of ERG recordings (eyes with episcleral celecoxib exoplant versus, intravitreal bevacizumab injection and control group) decreased during the study period. However, the intragroup comparison of the left and right eyes b-wave ratios at 8-week exam revealed no statistically significant differences (control group: *p* = 0.0672; intravitreal bevacizumab injection: *p* = 0.2887; episcleral celecoxib exoplant: *p* = 0.1700) (Table 2).

**Table 1:**
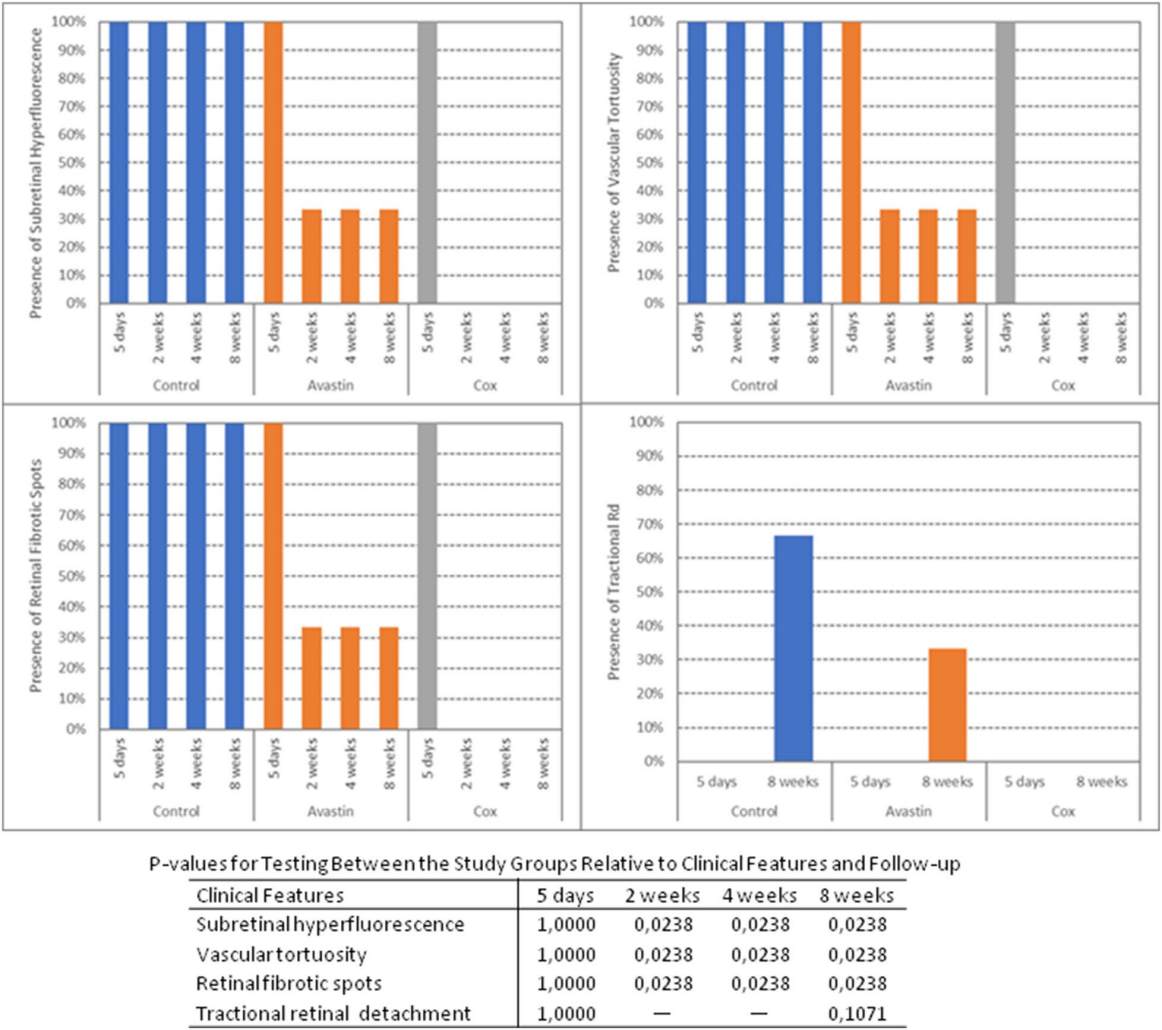
Frequency of clinical features in the study groups during follow-up period

**Table 2:** Mean b-wave amplitude ratios of electroretinography (ERG) recordings

	B-wave		
Control	OS	OD	% Function
9102	250.3	80.1	0.320016
9103	216.2	94.6	0.437558
9106	232.2	101.1	0.435401
Total	Avg		0.397658
	*p* value		0.067249
Avastin			
9105	227.5	34	0.149451
6104	117.8	63.1	0.535654
6117	144.9	103.5	0.714286
Total	Avg		0.466463
	*p* value		0.288704
Cox-2			
9112	251.4	97.9	0.389419
9115	230.2	165.7	0.719809
9109	219.9	106.6	0.484766
Total	Avg		0.531331
	*p* value		0.170046

*Avg* average

All the animals showed mild to moderate conjunctival swelling during the first four weeks of follow-up. The amount of conjunctival swelling gradually decreased and, at the end of follow-up (8 weeks) period, minimal anterior segment inflammation was visible. The same level of inflammatory reaction was also observed in both the anterior chamber and anterior vitreous during the first week following the surgical procedure. After this period, the inflammation progressively decreased and completely disappeared after 1 month of follow-up. Lens and cornea remained clear during the entire follow-up period, and the intraocular pressure remained normal, ranging from 12 to 18 mmHg in the study eyes. Extrusion of exoplant was not observed in the celecoxib exoplant group throughout the study follow-up.

## Discussion

Local intra-ocular delivery of drug has been a valuable approach of therapeutic development for retinal and choroidal diseases. Although repeated intraocular injections represent the current treatment for neovascular and proliferative retinal and choroidal diseases, they are an invasive method of treatment and do not provide sustained therapeutic drug levels in the retina and choroid [10]. In this setting, the development of new technology that allows local delivery in a manner that is less invasive and supply constant therapeutic drug levels in the retina and choroid is paramount. The transscleral delivery of drug using an episcleral exoplant has demonstrated selective drug diffusion through the sclera with minimal absorption by blood vessels in the periocular space [10]. There are two prototypes of episcleral exoplant that release drug slowly over time. One of these is most applicable for use with a solid sustained release formulation, such as a polymer, and the other prototype consists of a refillable exoplant that allows replenishment of drug within the device using liquid formulations or suspensions containing microspheres. Both prototypes of episcleral exoplant have been tested and showed diffusion of drug through the sclera resulting in high levels of drug in the retina and posterior vitreous [10].

In this study, we used a matrigel-based model to induce the development of retinal and choroidal neovascularization in rabbit eyes [12–14]. Intrinsic imaging features related to the retinal and choroidal neovascularization developed following matrigel subretinal injection, such as subretinal hyperfluorescence, retinal vascular tortuosity and retinal fibrotic spots, and were graded in regards to its persistence or regression during the follow-up study period to perform comparisons between the study groups (episcleral celecoxib exoplant, intravitreal bevacizumab injection and control group). We observed that there was a progressive regression of all three imaging features during the follow-up examinations that achieved a complete regression at 8-week examination in the episcleral celecoxib exoplant group. The cicatricial retinal traction that developed in one rabbit from the episcleral celecoxib exoplant at 8-week follow-up period may occur along with the regression of animal model CNV [15]. The involutional stage of CNV in an animal model is characterized by a decrease in cytokine production with associated retinal scarring and fibrosis [15], and, therefore, celecoxib may be related to cicatricial retinal traction since it reduces expression of inflammatory cytokines [16]. In the intravitreal bevacizumab injection group, although retinal vascular tortuosity disappeared in all three study rabbits, persistence of subretinal hyperfluorescence was observed in two rabbits and an extensive tractional retinal detachment occurred in one study rabbit. In all three study eyes from the control group, persistence of subretinal hyperfluorescence, retinal vascular tortuosity and retinal fibrotic spots was observed during and at the end of the follow-up period of 8 weeks. Regarding retinal toxicity, the mean b-wave amplitude ratios of ERG recordings at 8-week examination did not reveal statistically significant differences between the study groups.

VEGF is believed to be a major growth factor responsible for endothelial cells proliferation and neo-angiogenesis in neovascular AMD [17]. Since VEGF is a critical stimulus for both retinal and choroidal neovascularization, the development of VEGF antagonists has emerged lately and its intravitreal approach has been associated with visual acuity improvement in patients with neovascular AMD [3, 4]. Although the effect of intravitreous injection of VEGF antagonists is well-established, repeated intraocular injections represent an invasive therapeutic approach and do not provide sustained therapeutic drug levels in the retina and choroid [10].Also, the protection against angiofibrotic response by anti-VEGF agents is ephemeral since bevacizumab decreases the levels of connective tissue growth factor (CTFG) briefly in the first 3 to 6 days after intravitreal injection. CTFG is associated with angiofibrosis and consequent development of fibrovascular membranes and tractional retinal detachment in eyes with diabetic retinopathy [18]. In addition, CTFG has been related to CNV as well. Some reports have suggested that transdifferentiated RPE cells and vascular cells possess remarkable CTGF expression in CNVs [19, 20]. High levels of CTFG following anti-VEGF treatment could explain the appearance of tractional retinal detachment that occurred in one rabbit from the intravitreal bevacizumab injection group.

In the present study, we explored an alternative way to deliver intraocular celecoxib and demonstrated that the transscleral diffusion of celecoxib delivered from silicone episcleral exoplants caused complete regression of retinal and choroidal neovascularization created by a matrigel-based model in rabbit eyes. This regression of neovascularization occurred without the development of tractional retinal detachment due to angiofibrotic response. The complete regression of retinal and choroidal neovascularization following the placement of celecoxib exoplant is probably related to the Cox-2 inhibitor anti-VEGF and anti-proliferative effects on choroid-retinal endothelial cells and retinal pigment epithelial cells [21, 22]. It has been previously demonstrated that celecoxib inhibits VEGF secretion from retinal pigment epithelium (RPE) cells and has an anti-proliferative effect in the choroid-retinal endothelial cells [21, 22]. Therefore, celecoxib may have a dual beneficial effect by inhibiting VEGF secretion from RPE cells and preventing proliferation of endothelial cells. The statistically significant inhibition in VEGF secretion from ARPE-19 cells is obtained with celecoxib concentrations in the nanomolar range (10 nM or higher) [22], and the concentration required for statistically significant anti-proliferative effects of celecoxib on ARPE-19 cells is much higher (approximately 5 µM). In addition, the slope for VEGF inhibition is much shallower than that for the antiproliferative effects. The possible mechanisms for the anti-proliferative effect in endothelial cells by celecoxib could be inhibition of Akt signaling and apoptosis in the endothelial cells through interaction with extracellular signal-regulated kinase 2 (ERK2) and endoplasmic reticulum calcium ATPase signaling pathways [23, 24]. Celecoxib with its anti-inflammatory and anti-VEGF effects may provide a strong rationale for potential use itself or as an adjunct to currently existing therapies in the proliferative disorders of the eye, such as diabetic retinopathy and AMD. Our study does have some limitations such as the small number of rabbits in each study group that precluded a more powerful statistical analysis. Therefore, additional larger studies are needed to optimize sustained intraocular celecoxib delivery and to try to translate these findings into practical benefits for patients.

## Conclusions

Transscleral celecoxib delivery was demonstrated to significantly inhibit neovascularization in the study rabbit model of retinal and choroidal neovascularization. Although not statistically significant, an apparent effect of episcleral celecoxib on preventing tractional retinal detachment due to epiretinal fibrovascular proliferation was also observed. The episcleral celecoxib exoplant combined with sustained-release strategy may have impact in the treatment of retinal and choroidal proliferative diseases.

## Abbreviations

AMD: age-related macular degeneration
ARPE: adult retinal pigment epithelial
bFGF: basic fibroblast growth factor
COX: cyclooxygenase
CTFG: connective tissue growth factor
ERG: electroretinography
FA: fluorescein angiography
ISCEV: International Society for Clinical Electrophysiology of Vision
NPB: neomycin/polymyxin/bacitracin
OCT: optical coherence tomography
PBS: phosphate buffered saline
PLGA: poly (lactic-co-glycolic) acid
VEGF: vascular endothelial growth factor
