# Correction to: Sustained and targeted episcleral delivery of celecoxib in a rabbit model of retinal and choroidal neovascularization

**DOI:** 10.1186/s40942-018-0141-z

**Published:** 2019-01-07

**Authors:** Luiz H. Lima, Michel E. Farah, Glenwood Gum, Pamela Ko, Ricardo A. Pontes de Carvalho

**Affiliations:** 1 Federal University of Sao Paulo, Rua Botucatu, 821, Vila Clementino, São Paulo, SP CEP: 04023-062 Brazil; 2 Biological Test Center, Irvine, CA USA; 3 3T Ophthalmics, Irvine, CA USA

## Correction to: Int J Retin Vitr (2018) 4:31 10.1186/s40942-018-0131-1

Following publication of the original article [1], the authors reported the following changes to the article:

1. The correct name of Dr. De Carvalho is Ricardo A. Pontes de Carvalho.
2. The corresponding author of the article has changed to Ricardo A. Pontes de Carvalho.
  Email: rcarvalho@3tophthalmics.com
